# Identification of Food-Derived Electrophilic Chalcones as Nrf2 Activators Using Comprehensive Virtual Screening Techniques

**DOI:** 10.3390/antiox14050546

**Published:** 2025-04-30

**Authors:** Bingyu Bai, Piaohan Tu, Jiayi Weng, Yan Zhang, Quan Lin, Mitchell N. Muskat, Jie Wang, Xue Tang, Xiangrong Cheng

**Affiliations:** 1 School of Food Science and Technology, Jiangnan University, Wuxi 214122, China; 6220112001@stu.jiangnan.edu.cn (B.B.); 6230112116@stu.jiangnan.edu.cn (Y.Z.); 6220111198@stu.jiangnan.edu.cn (Q.L.); 8202407038@jiangnan.edu.cn (J.W.); tangxue@jiangnan.edu.cn (X.T.); 2 State Key Laboratory of Food Science and Resources, Jiangnan University, Wuxi 214122, China; 3 Beilun Market Supervision Administration, Ningbo 315800, China; 4 Michigan Medicine, University of Michigan, Ann Arbor, MI 48109, USA; mitchemu@umich.edu

**Keywords:** electrophilic compounds, machine learning, molecular docking, Nrf2 activators, chalcones

## Abstract

Electrophilic compounds are bioactive components commonly found in foods that are capable of covalently modifying nucleophilic sites on biologically functional macromolecules. These compounds may elicit positive bioactivity or negative biotoxicity, posing significant challenges in terms of time and resource expenditure in the de novo characterization of their biological activity. In this study, we developed a database of 332 food-derived electrophilic compounds and used a semi-supervised k-nearest neighbors (KNN) machine learning model to predict their bioactivity. Molecular docking analysis identified the three chalcone compounds with the highest potential positive activity—4-hydroxyderricin (4HD), isoliquiritigenin (ISO), and butein. Furthermore, in cell experiments, treatment with 4HD, ISO, and butein significantly reduced reactive oxygen species (ROS) levels. An RT-qPCR analysis demonstrated that these chalcones significantly upregulated the mRNA expression of *Nrf2* and its downstream antioxidant genes, including *Nqo1*, *HO-1*, *Gsr*, *Gclc*, and *Gclm*. ISO’s cytoprotective and antioxidant effects were abolished following these findings, which highlight that 4HD, ISO, and butein are effective Nrf2 activators and suggest that comprehensive virtual technology is a promising strategy for identifying functional bioactive compounds.

## 1. Introduction

Electrophilic compounds consist of small molecules featuring electrophilic functional groups capable of establishing covalent interactions with the nucleophilic components in biological macromolecules, including the thiols of cysteine residues in proteins or the guanine bases within DNA [1]. By covalently modifying critical cysteine thiols in redox-regulatory proteins, these compounds activate defense mechanisms against oxidative stress and inflammation, generating positive bioactivity (anti-inflammatory, anticancer, and neuroprotective properties) [2]. For instance, curcumin reversibly interacts with the Cys151 residue of Kelch-like ECH-associated protein 1 (Keap1), leading to the stabilization and enhanced nuclear translocation of Nuclear Factor Erythroid 2-Related Factor 2 (Nrf2). This activation subsequently triggers the expression of phase II metabolic enzymes and safeguards PC12 cells against the oxidative damage induced by H_2_O_2_ [3]. However, some electrophilic compounds, like acrolein, exhibit negative biotoxicity due to their non-selective alkylation of functional proteins and DNA. These irreversible covalent modifications can induce oxidative stress, cellular dysfunction, and disease development [4,5,6]. Therefore, it is essential to determine whether food-derived electrophilic compounds exert cytoprotective effects or pose potential toxicity to ensure their safe application as functional ingredients.

Food-derived electrophilic compounds typically contain structural motifs such as α, β-unsaturated carbonyls, isothiocyanates, ternary epoxides, disulfide bonds, and other characteristic structural groups [7]. Chalcones are naturally occurring electrophilic compounds found in various medicinal and edible plants [8]. Structurally, chalcones feature a conjugated planar framework comprising two benzene rings interconnected via a trans-α, β-unsaturated carbonyl moiety, making them highly efficacious as Michael acceptors [9].

Due to their low toxicity and notable efficacy, natural chalcones derived from medicinal plants have shown significant potential in drug development. Some chalcone-based drugs, such as sofalcone, are already clinically used for their gastroprotective properties [10,11]. Similarly, food-derived chalcones exhibit functional properties, including xanthoangelol from *Angelica keiskei*, which has hypoglycemic activity [12], and dihydrochalchalones such as phlorizin, trilobatin, and phloretin from *Lithocarpus polystachyus rehd* (Sweet tea), which possess antioxidant and anticancer activities [13]. Given their diverse bioactivities, investigating food-derived chalcones as electrophilic compounds targeting specific proteins is of great interest.

Nrf2 serves as a pivotal transcription factor responsible for regulating redox homeostasis [14]. Under physiological conditions, Keap1 negatively regulates Nrf2 by facilitating its ubiquitination and subsequent degradation, thereby maintaining low cellular Nrf2 levels [15]. When oxidative stress occurs, Nrf2 dissociates from its repressor Keap1. This dissociation allows Nrf2 to translocate to the nucleus and activates protective antioxidant genes [16,17]. The activation of the Nrf2 pathway has gained recognition as a potential therapeutic strategy for preventing and managing diseases like cancer, Alzheimer’s disease, and Parkinson’s disease [18,19]. Traditional Nrf2 activators, including oxidizable phenols, quinones, and dithiols, primarily function by interacting with Keap1’s cysteine sulfhydryl groups [20,21]. However, these compounds often exhibit off-target effects by interacting with other cysteine-containing proteins, leading to unintended physiological consequences [22]. Since food-derived compounds are generally safe and bioavailable, they offer a promising alternative for Nrf2 activation with fewer off-target effects.

Advancements in bioinformatics have enabled the rapid discovery and optimization of bioactive compounds [23]. Machine learning, an essential tool in computational drug design, builds predictive models using large datasets to identify potential functional molecules [24]. These models efficiently process high-dimensional data, uncover hidden patterns, and generalize predictions to new compounds [25]. In functional compound development, machine learning has been applied to target recognition, small-molecule optimization, biomarker selection, and diagnostic imaging [26,27]. Protein–ligand interactions are critical in cellular processes such as signal transduction and gene regulation [28]. Virtual screening, a computational technique, evaluates large compound libraries by simulating their binding to target proteins [29,30]. By analyzing physicochemical properties and ranking compounds based on their binding affinity, virtual screening accelerates the identification of promising lead compounds [31]. This method is widely used to predict compound–target interactions and elucidate mechanisms of action efficiently.

While electrophilic compounds can exert beneficial effects such as antibacterial, anti-inflammatory, and antioxidant activities through covalent modifications, they can also pose a toxicity risk by irreversibly binding to biological macromolecules, leading to oxidative damage and disease development. To address these concerns, this study established a database of food-derived electrophilic compounds, used machine learning techniques to rapidly screen for bioactive candidates, and utilized molecular docking to identify potential Nrf2 activators. The cytoprotective effects of selected compounds were further validated using oxidative damage cell models, providing insights into their potential as functional bioactive ingredients.

## 2. Materials and Methods

### 2.1. Materials and Chemicals

HepG2 cells were acquired from the Cell Resource Center of the Shanghai Institute of Biological Sciences, China. Isoliquiritigenin (ISO, 98%) and butein (98%) were purchased from McLean & Co. (Shanghai, China), while 4-hydroxyderricin (4HD, 98%) was obtained from Kunming Plant Fraction Co., Ltd. (Kunming, China). Dextran sulfate sodium salt was supplied by McLean & Co. (Shanghai, China). Cell culture reagents, including DMEM medium, Opti-MEM medium, and PBS buffer, were sourced from Hyclone Co. (Logan, UT, USA). Additional reagents included RIPA lysis buffer (strong), a gel rapid preparation kit, and a nuclear and cytoplasmic protein separation kit, all from Biyuntian Co., Ltd. (Shanghai, China). Biochemical analyses were conducted using antioxidant and oxidative stress assay kits, which included measurements of total antioxidant capacity (T-AOC), catalase (CAT) activity, superoxide dismutase (SOD) activity, glutathione peroxidase (GSH-Px) activity, malondialdehyde (MDA) levels, and myeloperoxidase activity, and an active oxygen detection kit.

For molecular biology experiments, MonScript™ RTIII All-in-One Mix Reverse Transcription Kit, Lipo8000™ transfection reagent, and SYBR Green qPCR Mix Kit were obtained from Mona Biotechnology Co., Ltd. (Suzhou, China), while target gene primers were synthesized by Anshengda Biotechnology Co., Ltd. (Suzhou, China). Antibodies, including β-actin, Nrf2, Nqo1, HO-1, Lamin B1, GAPDH, and rabbit secondary antibodies, were procured from the CST Corporation (Houston, TX, USA).

Experimental equipment included an SW-CJ-1F ultra-clean workbench from Suzhou Instrument Factory No. 4 (Suzhou, China), a Countess Type II automatic cell counter, a Forma Series 3WJ CO_2_ incubator, and a Nanodrop 2000 spectrophotometer from Thermo Scientific (Waltham, MA, USA). Other instruments included a multifunctional enzyme marker from Biotek (Seattle, WA, USA), a wide-field high-content cell imaging system manufactured by Molecular Devices (San Jose, CA, USA), and a real-time fluorescence quantitative PCR instrument from Suzhou Monad Company (Wuhan, China).

### 2.2. Machine Learning

Using the structural characteristics of electrophilic compounds, a systematic selection and screening process was conducted across four major databases: the TOXNET Toxicology Database (National Library of Medicine, NLM, Bethesda, MD, USA), the Comparative Toxicogenomics Database (CTD) established by the National Institute of Environmental Health Sciences (NIEHS) at North Carolina State University, the Compound Reference Database of the Chinese Academy of Sciences, and the Toxicology Research Database of the Chinese Academy of Sciences. This process aimed to identify food-derived electrophilic compounds.

Each electrophilic compound identified had its corresponding Chemical Abstracts Service (CAS) number utilized to retrieve information pertaining to its food sources from PubChem (https://pubchem.ncbi.nlm.nih.gov/, 23 February 2022). Subsequently, the 3D structures of these compounds were downloaded in .SDF format and processed through the Dragon website (http://www.talete.mi.it/index.htm, 23 February 2022) to compute molecular descriptors, which quantitatively encapsulate structural, physicochemical, and electronic properties. This analysis generated 1644 molecular descriptors, forming a comprehensive database of food-derived electrophilic compounds. From this dataset, a subset of 332 compounds was selected for further investigation.

To develop predictive models, the Scikit-Learn machine learning platform in Python (https://www.python.org/, 25 February 2022) was utilized. Four classification algorithms—logistic regression, support vector machine (SVM), k-nearest neighbor (KNN), and decision tree—were trained using a feature-selected dataset. The optimal model was determined through parameter tuning and subset validation, achieving the highest accuracy and recall rates for predicting the bioactivity of food-derived electrophilic compounds.

### 2.3. Molecular Docking and Virtual Screening

The 3D structures of the Keap1 BTB domain (PDB ID: 4CXI) and the Kelch domain (PDB ID: 2FLU) were retrieved from the Protein Data Bank (PDB) (https://www.pdbus.org/, 25 February 2022). AutoDock Tools (ADT, version 1.5.6) were utilized to append polar hydrogen atoms and assign Gasteiger charges to both the ligand compounds and the protein structures. Molecular docking studies were conducted using the following grid coordinates: Kelch domain (x = −3.0, y = 19.2, z = 2.3) and BTB domain (x = 5.4, y = 5.0, z = −9.4). Affinity values and docking scores were used to determine the optimal ligand binding poses, with the complex exhibiting the lowest free binding energy selected for further analysis.

In order to refine their molecular structure, the lowest energy conformation of the electrophilic compounds derived from food sources was optimized using Gaussian 09 software (Gaussian Inc., Wallingford, CT, USA). This optimization was conducted at the DFT/B3LYP/6-311G+(d,p) level of theory. The optimized structures were then analyzed using Multiwfn 3.2 (Beijing Kein Research Center for Natural Sciences, Beijing, China) to enable wave function calculations and evaluate the electrophilic properties of the compounds.

### 2.4. Cell Culture

Endogenous reactive oxygen species such as hydrogen peroxide (H_2_O_2_) are significant contributors to oxidative damage [32]. Given that excessive oxidative stress readily impairs hepatic tissues, this study employs an H_2_O_2_-induced oxidative injury model of HepG2 cells to evaluate the cytoprotective effects of electrophilic compounds [33]. HepG2 cells were cultivated in DMEM medium enriched with 10% fetal bovine serum (FBS) and 1% penicillin–streptomycin solution within a 37 °C incubator maintained at 5% CO_2_. Experiments were conducted when the cells reached approximately 80% confluency. To establish an oxidative stress model, the cells were subjected to different concentrations of H_2_O_2_, ranging from 0.2 mM to 1.5 mM (specifically, 0.2 mM, 0.4 mM, 0.6 mM, 0.8 mM, 1.0 mM, 1.2 mM, and 1.5 mM), for a duration of 4 h. The CCK-8 assay was employed to evaluate cell viability, thereby determining the optimal concentration of H_2_O_2_ necessary to induce oxidative stress in HepG2 cells.

### 2.5. Determination of Antioxidant Biochemical Indexes

In accordance with previous studies, cells were plated in 96-well transparent-bottom black plates and allowed to incubate for a period of 24 h [34]. After the initial incubation period, the cells were treated with 0.6 mM H_2_O_2_ for 4 h to induce oxidative stress. Subsequently, they were incubated in a dark medium containing a 2′,7′-dichlorofluorescein diacetate (DCFH-DA) fluorescent probe. After washing with PBS, intracellular reactive oxygen species (ROS) levels were visualized using wide-field high-content imaging. Additionally, biochemical assays were performed to measure catalase (CAT), total antioxidant capacity (T-AOC), superoxide dismutase (SOD), glutathione peroxidase (GSH-Px), and malondialdehyde (MDA) levels, following their respective assay kit instructions, to assess the oxidative stress status and antioxidant defenses of the cells.

### 2.6. Real-Time Fluorescence Quantitative PCR

Total RNA was extracted from cells through the utilization of Trizol. RNA quality was checked with a NanoDrop. Then, 1 μg of RNA was converted to cDNA. For RT-qPCR analysis, the cDNA was combined with SYBR Green Mix to prepare a 20 μL reaction mixture. Gene expression levels were quantified using a 7500 Fast PCR system. The relative mRNA expression was calculated using the 2^−ΔΔCt^ method, with β-actin as the internal control. The sequence of primers was shown in Table 1.

### 2.7. Statistical Analysis

Statistical analyses were performed using GraphPad Prism 8.1 and SPSS 23.0. For both univariate and bivariate analyses of variance (ANOVAs), a general linear model was applied, while pairwise comparisons were conducted using the least significant difference (LSD-t) test. The experimental outcomes were reported as means ± standard deviation (SD), with statistical significance established at *p* < 0.05.

## 3. Results

### 3.1. Machine Learning Analysis of Food-Derived Electrophilic Compounds

**Figure 1:**
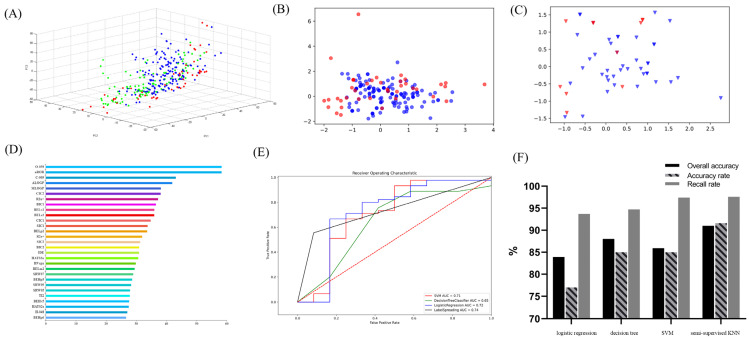
Machine learning analysis of food-derived electrophilic compounds. (**A**) Principal component analysis of dataset. (**B**) Principal component analysis of training set. (**C**) Principal component analysis of test set (blue represents positive directivity, red represents negative directivity, and green represents uncertainty). (**D**) The F-values of the descriptors. (**E**,**F**) ROC curves of logistic regression, support vector machine, decision tree, and k-nearest neighbor machine learning models.

A food-derived electrophilic compounds database comprising 332 compounds was utilized to classify the biological properties of electrophilic compounds found in food sources. By analyzing their three-dimensional structures, a total of 1664 molecular descriptors were identified. To reduce the dimensionality of this dataset and transform high-throughput data into intuitively readable 2D or 3D models, a principal component analysis (PCA) was applied. As shown in Figure 1A–C, the blue balls representing positive effects and the red balls representing negative effects are stacked on top of each other with no obvious boundaries. Besides, The green balls represent foodborne electrophilic compounds of unknown potency. This situation calls for the use of more machine learning models, which utilize training datasets, to predict the biological activities of these compounds.

Feature extraction, a crucial step in image analysis and data processing, was performed using the “f_classif” function in the Scikit-Learn (https://scikit-learn.org/stable/, 8 February 2022) machine learning platform to compute F-values. As shown in Figure 1D, while a single descriptor could provide a basic correlation between the positive and negative bioactivities of electrophilic compounds, it was insufficient for capturing deeper relationships, necessitating the use of a more detailed descriptor analysis.

To develop an accurate prediction model, four machine learning models were employed: logistic regression, decision tree, SVM, and a semi-supervised KNN. The models were evaluated using several key performance metrics, such as accuracy, precision, recall, and the receiver operating characteristic (ROC) curve. The area under the ROC curve (AUC) served as the metric used to evaluate the overall performance of the models, with values approaching 1 signifying superior predictive accuracy. As depicted in Figure 1E,F, the semi-supervised KNN model outperformed the other models and was therefore selected for the statistical analysis of the test dataset.

**Figure 2:**
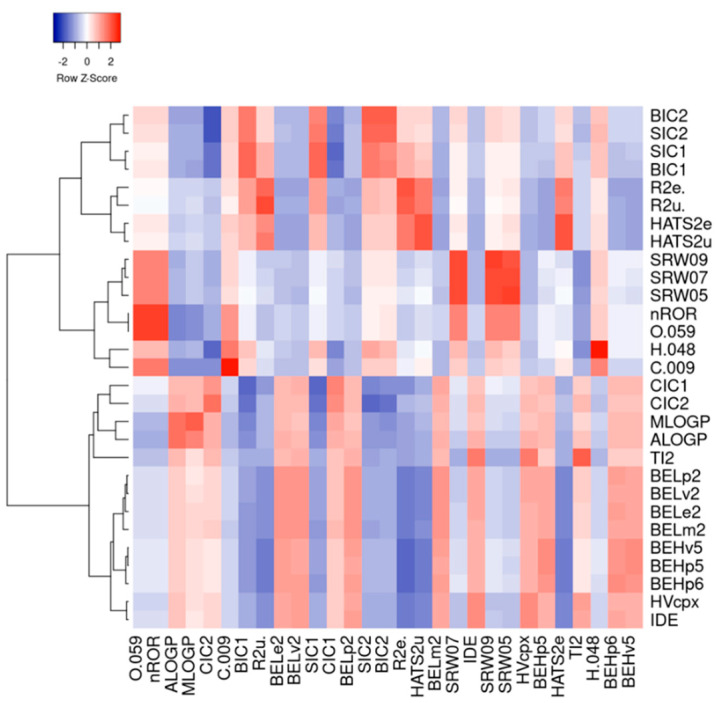
Clustering heat map based on eigenvalue correlation coefficient matrix.

Furthermore, a hierarchical cluster analysis was conducted based on the correlation coefficients of the top 2% of eigenvalues from the F-value calculations (Figure 2). This analysis revealed that group properties, electronegativity, and lipophilicity are key factors in determining the bioactivity of food-derived electrophilic compounds. A comprehensive dataset assessment indicated that negatively classified electrophilic compounds were more likely to contain aliphatic ether structures and heteroatoms, contributing to their higher electrophilicity. In contrast, positively classified electrophilic compounds exhibited greater lipophilicity (Figure S1). The parameter definitions of the corresponding eigenvalue names are provided in Table S1.

### 3.2. Virtual Screening of Cytoprotective Electrophilic Compounds

**Figure 3:**
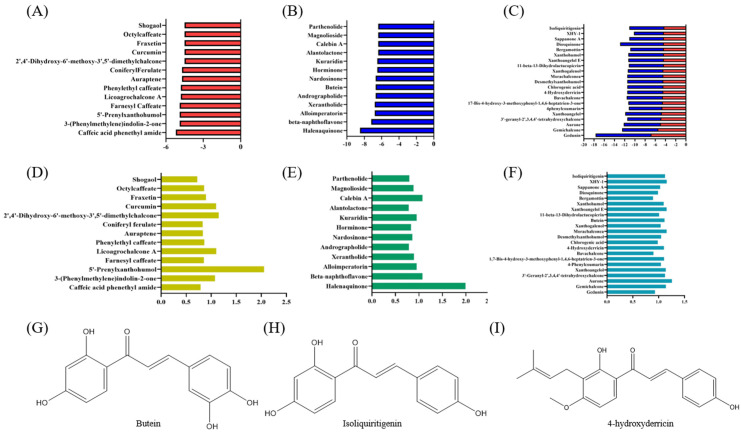
Virtual screening of cytoprotective electrophilic compounds. (**A**–**C**) Molecular docking scores. (**D**–**F**) Fukui function. (**G**–**I**) Structure of butein, isoliquiritigenin, and 4-hydroxyderricin.

This study used AutoDock Vina (25 February 2022) to perform molecular docking and evaluate the docking conformation scores of compounds with the Keap1 protein for virtual screening (Figure 3A–C). The docking results, combined with a Fukui function analysis (Figure 3D–F), revealed significant differences in the binding affinities of various compounds with Keap1. Notably, the overall affinity scores and the proportion of chalcones among the cross-hit molecules interacting with Cys434 were higher than those interacting with Cys151.

Among the identified chalcones, 4-hydroxyderricin (4HD) from *Angelica keiskei*, isoliquiritigenin (ISO) from *Glycyrrhiza*, and butein from *Cashew* demonstrated substantially stronger binding affinities compared to sulforaphane, a well-characterized electrophilic Nrf2 activator [35]. These three chalcones were selected from the cross-hit molecules due to their high binding energy and Fukui function scores. Covalent docking analysis further demonstrated that these compounds can form covalent bonds with the cysteine thiol groups of Keap1 at Cys151 and Cys434, facilitated by their α, β-unsaturated carbonyl groups.

**Figure 4:**
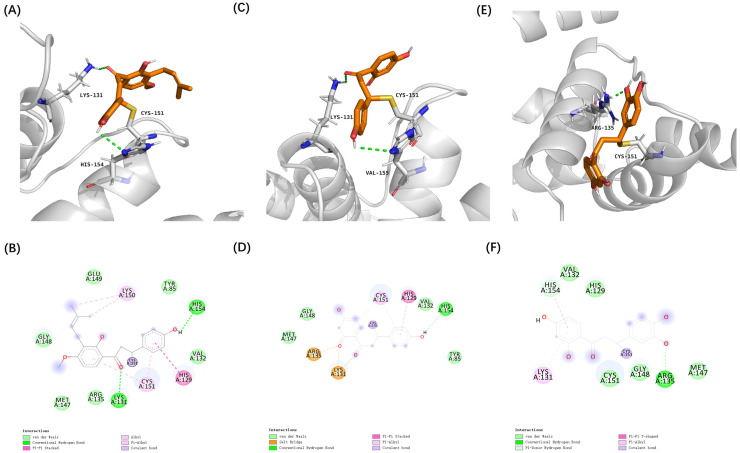
Covalent docking poses of cytoprotective electrophilic compounds and Cys151 of Keap1. (**A**,**B**) 4HD. (**C**,**D**) ISO. (**E**,**F**) butein.

**Figure 5:**
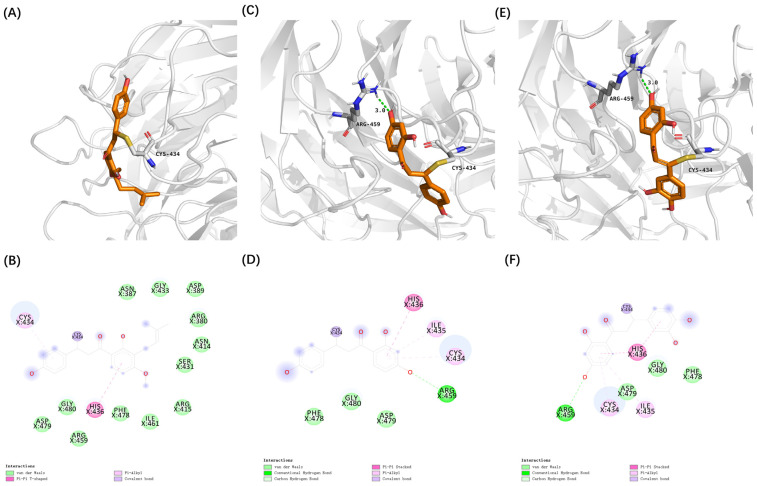
Covalent docking poses of cytoprotective electrophilic compounds and Cys434 of Keap1. (**A**,**B**) 4HD. (**C**,**D**) ISO. (**E**,**F**) butein.

Furthermore, these covalent interactions were supported by secondary interactions, such as hydrogen bonding and van der Waals forces. These secondary interactions can lead to conformational changes within the Keap1 protein (Figure 4 and Figure 5). These findings suggest that 4HD, ISO, and butein have the potential to activate the Nrf2 signaling pathway, which is a key cellular defense mechanism against oxidative stress.

### 3.3. Verification of the Protective Effect of Chalcones on Oxidative-Damaged Cell Models

H_2_O_2_ has been widely reported to induce oxidative damage in HepG2 cells by suppressing the expression of Nrf2 and its downstream antioxidant proteins. In this study, we established a H_2_O_2_-induced oxidative stress model in HepG2 cells to investigate the Nrf2-activating potential of 4HD, ISO, and butein [36,37]. As shown in Figure S2A,B, incubation with 0.6 mM H_2_O_2_ for 4 h did not significantly affect cell viability, but it led to a significant increase in intracellular ROS levels (*p* < 0.05). Based on these findings, this condition was used to establish an oxidative damage model for evaluating the cytoprotective effects of 4HD, ISO, and butein.

**Figure 6:**
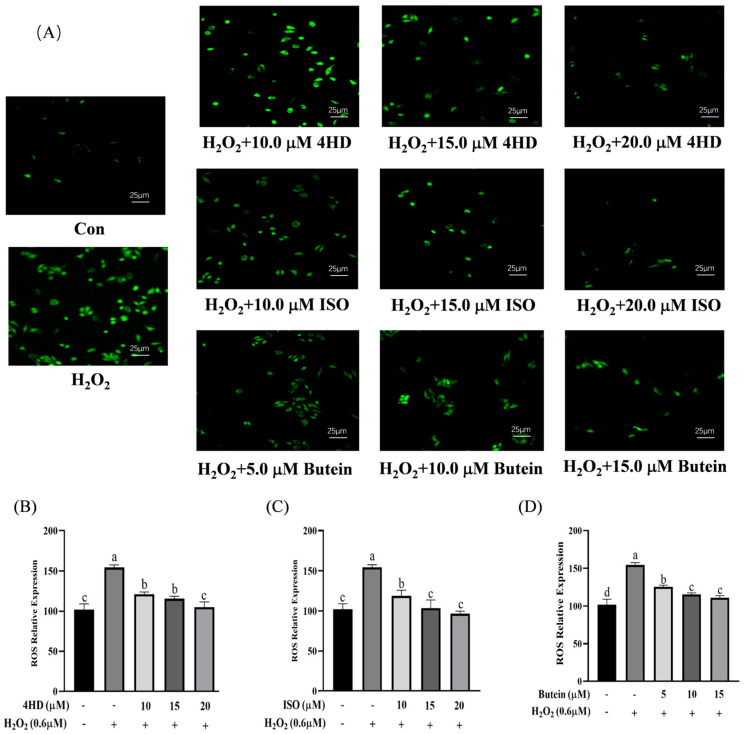
Effect of three compounds on ROS production in HepG2 cells. (**A**) Fluorescence imaging diagrams at 20× magnification. (**B**–**D**) ROS relative expression in 4HD, ISO, and butein group. Different lowercase letters represent a statistically significant difference.

HepG2 cells were incubated with gradient concentrations of 4HD, ISO and butein for 24 h to determine their appropriate experimental concentrations, and cell survival rates were measured (Figure S2C–E). After treatment with the three chalcones, fluorescence intensity was observed using a wide-field high-content imaging system with the FITC fluorescence channel (Figure 6A). H_2_O_2_ exposure induced a notable increase in intracellular ROS levels, whereas 4HD significantly reduced ROS levels in a dose-dependent manner (Figure 6B). H_2_O_2_-treated cells exhibited the highest green fluorescence intensity due to the large number of free radicals produced. Following treatment with different concentrations of 4HD, their fluorescence intensity was effectively reduced, consistent with the quantitative analysis. The results indicate that 4HD has a potent inhibitory effect on the production of intracellular ROS.

Similarly, ISO and butein also demonstrated potent ROS-scavenging activity, significantly improving redox-related indicators after the cells were incubated for 24 h (Figure 6C,D). These findings indicate that 4HD, ISO, and butein exhibit strong antioxidant properties and may effectively mitigate oxidative stress-induced cellular damage.

**Figure 7:**
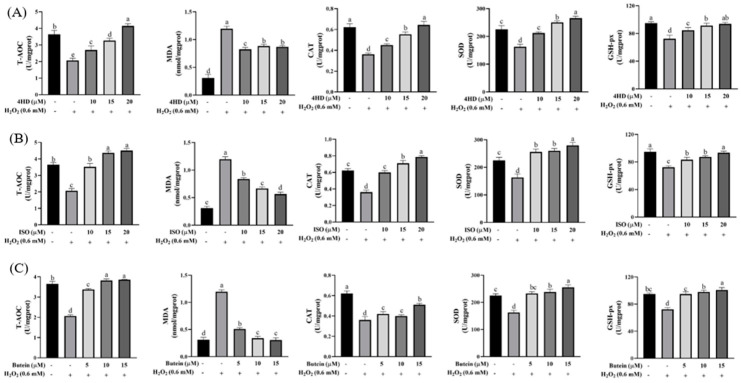
Effects of three electrophilic compounds on the activities of T-AOC, MDA, CAT, SOD, and GSH-Px in H_2_O_2_-induced oxidative stress. (**A**–**C**) Activities of T-AOC, MDA, CAT, SOD, and GSH-Px in 4HD, ISO, and butein group. Different lowercase letters represent a statistically significant difference.

As shown in Figure 7, treatment with 4HD, ISO, and butein significantly enhanced the activities of SOD, CAT, and GSH-Px, which were otherwise reduced by H_2_O_2_ exposure. Additionally, these compounds increased T-AOC levels, decreased MDA content, and exhibited a dose-dependent effect (*p* < 0.05).

For instance, with a low dose (10 μM) of 4HD, antioxidant enzyme activity was significantly higher than in the H_2_O_2_-treated group (*p* < 0.05). Further increasing the dose to 20 μM led to an even greater enhancement of antioxidant enzyme activity (*p* < 0.05). These findings indicate that 4HD, ISO, and butein effectively counteract oxidative stress by boosting cellular antioxidant defense mechanisms in a dose-dependent manner.

### 3.4. Expression Analysis of Nrf2 Signaling Pathway-Related Genes

**Figure 8:**
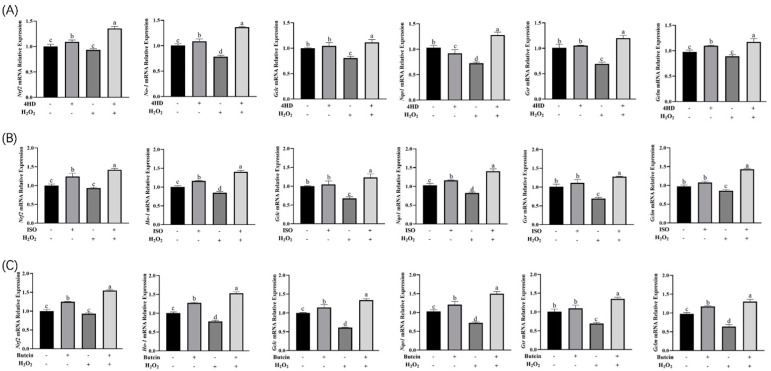
Effects of three electrophilic compounds on the expression of Nrf2 signaling pathway-related genes: *Nrf2*, *HO-1*, *Gclc*, *Nqo1*, *Gsr*, and *Gclm*. (**A**–**C**) Relative expression of Nrf2 pathway related genes in 4HD, ISO, and butein group. Data with different lowercase letters were significantly different (*p* < 0.05).

As shown in Figure 8, H_2_O_2_ exposure significantly reduced the expression of Nrf2 and its downstream genes in HepG2 cells. However, treatment with 4HD led to a notable upregulation of *Nrf2*, *Nqo1*, *Gclm*, *Gsr*, *Gclc*, and *HO-1* mRNA levels, with pronounced increases for *Nrf2*, *Gclm*, and *HO-1*. Similarly, following H_2_O_2_-induced oxidative stress, treatment with ISO or butein significantly increased the expressions of *Nrf2*, *Nqo1*, *Gclm*, *Gsr*, *Gclc*, and *HO-1* (*p* < 0.05), suggesting a potential link between the antioxidant effects of ISO and butein and their regulation of the *Nrf2-ARE* pathway. These findings suggest that 4HD, ISO, and butein alleviate oxidative stress by upregulating *Nrf2* expression and its downstream targets, including *Nqo1*, *Gclm*, *Gsr*, *Gclc*, and *HO-1*. This activation enhances endogenous antioxidant enzyme expression and prevents the accumulation of ROS, thereby protecting cells from oxidative damage.

## 4. Discussion

Food-derived electrophilic compounds exhibit diverse physiological activities, particularly in modulating oxidative stress. They exert antioxidant effects by modifying Keap1, facilitating the nuclear translocation of Nrf2. However, some electrophilic compounds can bind non-specifically to other protein sites, leading to hepatorenal toxicity. To address this concern, this study used machine learning and molecular docking to identify non-toxic food-derived electrophilic compounds, ultimately identifying three chalcones (4HD, ISO, and butein) with potential Nrf2-activating properties. Their ability to activate Nrf2 and provide cytoprotection against oxidative stress was subsequently validated through cell-based experiments, offering a novel approach to the screening of bioactive food-derived electrophilic compounds.

Electrophilic compounds readily undergo covalent interactions with electron-rich biomacromolecules, leading to them having diverse bioactive roles [38,39]. Natural electrophilic compounds such as curcumin, hesperidin, and lycopene have demonstrated cytoprotective effects by activating the farnesoid X receptor (FXR) pathway and regulating the Nrf2 pathway [40]. However, the dual nature of electrophilic compounds—exhibiting both cytoprotective and cytotoxic effects—poses a challenge for their practical application [41]. As a result, extensive resources have been dedicated to identifying the potential functions of electrophilic compounds to avoid the undesired cytotoxicity of food-derived electrophilic compounds after their supplementation.

Virtual screening integrates machine learning and molecular docking to accelerate electrophilic compound discovery. Supervised machine learning algorithms, including logistic regression (probabilistic classification via sigmoid-transformed linear decision boundaries) and decision trees (hierarchical feature splitting via recursive partitioning), enable binary activity prediction for labeled datasets, while support vector machines (SVMs) with kernel-based separation excel in modeling high-dimensional quantitative structure–activity relationships (QSARs) with limited samples [42]. Semi-supervised k-nearest neighbors (KNN) leverage both labeled/unlabeled data for scaffold-hopping predictions, making them particularly effective in data-scarce scenarios [43]. Molecular docking with AutoDock Vina predicts binding poses through empirical force field-based scoring functions, prioritizing compounds with optimal target complementarity [44]. Machine learning models have been successfully employed for compound screening, such as in a study by Guttman et al., who used computer-based deep learning to identify CYP3A4 inhibitors from a dataset of 60,000 dietary compounds [45]. These results underscore the potential of computational approaches in distinguishing between the cytoprotective and cytotoxic roles of food-derived electrophilic compounds based on their unique structural features.

We developed a compound library containing 1644 molecular descriptors extracted using Dragon software 5.3 to advance this approach further. Semi-supervised learning techniques, such as the KNN model, were employed to analyze unannotated data efficiently, reducing computational costs. The semi-supervised KNN model outperformed logistic regression, decision tree, and SVM models, demonstrating the highest predictive accuracy [46].

Nrf2 is key in combating oxidative stress [47]. Investigating whether electrophilic compounds activate the Nrf2-Keap1 pathway and their potential mechanisms can provide new strategies for combating oxidative stress-related diseases. Molecular docking, a computational technique used for evaluating ligand–protein interactions, helps identify binding affinities while avoiding undesirable off-target effects [48].

Previous studies have reported that Cys151 and Cys434 are key sites for Nrf2 activation. Pubescenoside A, for example, selectively covalently binds to Keap1 at Cys77 and Cys434, thereby inhibiting Nrf2 ubiquitination and promoting phase II enzyme activation [49]. Additionally, covalent modification at Cys151 in the Keap1 BTB domain has been shown to enhance Keap1 ubiquitination and prolong the half-life of Nrf2 [50]. The reactivity of electrophilic compounds is influenced by both their covalent and non-covalent interactions, highlighting the importance of binding affinity and stability [51]. This study used molecular docking to screen the electrophilic compounds targeting Cys151 and Cys434, identifying three chalcones: 4HD, ISO, and butein. Structurally, chalcones contain an α, β-unsaturated carbonyl group, which is more likely to react with sulfhydryl-containing proteins rather than hard nucleophiles such as hydroxyl or amino groups in nucleic acids, reducing their carcinogenic and mutagenic risks [52,53].

H_2_O_2_-induced oxidative stress models were established in HepG2 cells to evaluate the antioxidant potential of these chalcones. H_2_O_2_ easily penetrates the cell membrane, producing excess ROS, which can lead to mitochondrial damage, oxidative imbalance, and the progression of various diseases, including atherosclerosis, neurodegeneration, and aging [54]. CCK-8 kit findings showed that 4HD and ISO (0–20 μM) did not exhibit significant cytotoxicity, while butein showed mild cytotoxic effects at 20 μM. Importantly, all three compounds significantly reduced intracellular ROS levels and increased the activity of antioxidant enzymes including SOD, CAT, and GSH-Px, which are essential for neutralizing oxidative stress [55]. Additionally, they reversed H_2_O_2_-induced MDA accumulation, further supporting their antioxidant efficacy.

The activation of Nrf2 and its downstream pathways is a fundamental cellular defense mechanism against ROS-induced damage [56]. Molecular docking confirmed that food-derived electrophilic chalcones can activate Nrf2, and subsequent cellular experiments validated their role in promoting Nrf2’s nuclear translocation [57]. It was shown that 4HD, ISO, and butein upregulated Nrf2 and its downstream genes, including HO-1, Nqo1, Gsr, Gclc, and Gclm, which play critical roles in antioxidant defense [58,59]. These results supported that 4HD, ISO, and butein can enhance cell antioxidant capacity by activating Nrf2 and its downstream antioxidant pathway, consistent with molecular docking predictions. Based on molecular docking, the substances with greater activation potential in these natural compounds were preliminarily screened, saving additional economic investment and the consumption of medical resources.

In a previous study, multiple food-derived electrophilic compounds were found to relieve DSS-induced colitis in mice by activating the Nrf2-Keap1 pathway, which revealed the potential of food-derived electrophilic compounds to act as Nrf2 activators [6]. In this study, we further screened three food-derived electrophilic compounds with the potential for Nrf2 activation by machine learning and molecular docking and verified their activity by cell experiments. While this study primarily focused on the covalent interactions between electrophilic compounds and Keap1/Nrf2, Nrf2 activation is also regulated by phosphorylation. Nrf2 contains multiple functional domains rich in serine (Ser), threonine (Thr), and tyrosine (Tyr) residues, which provide potential phosphorylation sites [60]. Recent studies suggest that AMPK phosphorylates Nrf2, promoting its nuclear translocation and participation in redox and energy homeostasis [61]. Additionally, protein kinase C (PKC) reduces Nrf2 ubiquitination via Ser phosphorylation, further enhancing its stability [62]. Given the significant role of phosphorylation in Nrf2 activation, future studies could employ machine learning techniques to explore Nrf2 phosphorylation mechanisms and optimize the identification of Nrf2 activators. Given the potential of oxidative stress to induce telomere shortening and DNA damage, further investigation is warranted to explore whether isoprenylated chalcones exhibit genoprotective effects [63].

## 5. Conclusions

Virtual screening represents a cost-effective and efficient strategy for identifying food-derived compounds that act as Nrf2 activators. In this study, we rapidly identified three food-derived electrophilic compounds, 4HD, ISO, and butein, through virtual screening techniques, including machine learning and molecular docking. All of these compounds, belonging to the chalcone class, exhibited strong potential as Nrf2 activators. Cell-based experiments confirmed that 4HD, ISO, and butein effectively mitigated H_2_O_2_-induced oxidative damage in HepG2 cells via Nrf2 pathway activation. These findings establish and validate a robust and efficient screening method for identifying food-derived electrophilic chalcones with potential Nrf2-activating and cytoprotective properties.

## Figures and Tables

**Table 1:** Sequences of the primers.

Gene	Forward Primer (5′-3′)	Reverse Primer (5′-3′)
*Nrf2*	TCCAGTCAGAAACCAGTGGAT	GAATGTCTGCGCCAAAAGCTG
*Nqo1*	GAAGAGCACTGATCGTACTGGC	GGATACTGAAAGTTCGCAGGG
*Gclc*	GGAGACCAGAGTATGGGAGTT	CCGGCGTTTTCGCATGTTG
*Gclm*	CATTTACAGCCTTACTGGGAGG	ATGCAGTCAAATCTGGTGGCA
*HO-1*	AAGACTGCGTTCCTGCTCAAC	AAAGCCCTACAGCAACTGTCG
*Gsr*	CACGAGTGATCCCAAGCCC	CAATGTAACCTGCACCAACAATG
*β-actin*	CATGTACGTTGCTATCCAGGC	CTCCTTAATGTCACGCACGAT

## Data Availability

The curated dataset of 332 food-derived electrophilic compounds is available in Supplementary Table S2.

## Supplementary Materials

The following supporting information can be downloaded at https://www.mdpi.com/article/10.3390/antiox14050546/s1. Figure S1: Differences in correlation descriptors for different features of electrophilic compounds; Table S1: Descriptions of the descriptors; Table S2: RMSD values of compound docking with Cys434; Table S3: RMSD values of compound docking with Cys151; Figure S2: Cell redox levels.
