# Supplementary material for: Identification of Food-Derived Electrophilic Chalcones as Nrf2 Activators Using Comprehensive Virtual Screening Techniques
Source: Antioxidants (Basel). 2025 Apr 30;14(5):546. doi: 10.3390/antiox14050546 (PMC12108417; doi:10.3390/antiox14050546)
Supplement: Supplementary file 1 [file antioxidants-14-00546-s001.zip › antioxidants-3604637-supplementary.pdf]

supplementary materials

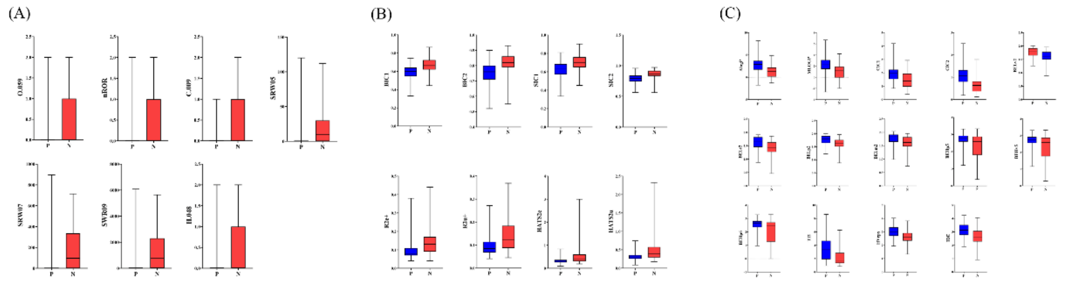

Figure S1. Differences in correlation descriptors for different features of electrophilic compounds. (A) group characteristics of electrophilic compounds. (B) electronegativity characteristics of electrophilic compounds. (C) lipophilicity characteristics of electrophilic compounds. P: Positive effect; N: Negative effect.

Table S1 The description of the descriptors

Abbreviation	Parameter description
O-059	Al-O-Al
nROR	number of ethers (aliphatic)
C-009	CHRX2
ALOGP	Ghose-Crippen octanol-water partition coeff. (LogP)
MLOGP	Moriguchi octanol-water partition coeff. (LogP)
CIC2	complementary information content (neighborhood symmetry of 2-order)
R2u+	R maximal autocorrelation of lag 2 / unweighted
BIC1	bond information content (neighborhood symmetry of 1-order)
BELv2	lowest eigenvalue n. 2 of Burden matrix / weighted by atomic van der Waals volumes
BELe2	lowest eigenvalue n. 2 of Burden matrix / weighted by atomic Sanderson electronegativities
CIC1	complementary information content (neighborhood symmetry of 1-order)
SIC1	structural information content (neighborhood symmetry of 1-order)

BELp2	lowest eigenvalue n. 2 of Burden matrix / weighted by atomic polarizabilities
R2e+	R maximal autocorrelation of lag 2 / weighted by atomic Sanderson electronegativities
SIC2	structural information content (neighborhood symmetry of 2-order)
BIC2	bond information content (neighborhood symmetry of 2-order)
IDE	mean information content on the distance equality
HATS2u	leverage-weighted autocorrelation of lag 2 / unweighted
HVcpx	graph vertex complexity index
BELm2	lowest eigenvalue n. 2 of Burden matrix / weighted by atomic masses
SRW07	self-returning walk count of order 07
BEHp5	highest eigenvalue n. 5 of Burden matrix / weighted by atomic polarizabilities
SRW09	self-returning walk count of order 09
SRW05	self-returning walk count of order 05
TI2	second Mohar index TI2
BEHv5	highest eigenvalue n. 5 of Burden matrix / weighted by atomic van der Waals volumes
HATS2e	leverage-weighted autocorrelation of lag 2 / weighted by atomic Sanderson electronegativities
H-048	H attached to C2(sp3)/C1(sp2)/C0(sp)
BEHp6	highest eigenvalue n. 6 of Burden matrix / weighted by atomic polarizabilities

Table S2 RMSD value of compound docking with Cys434

Compounds	RMSD l.b	RMSD u.b
Gedunin	1.148	3.981
Halenaquinone	1.28	1.785
Diosquinone	1.598	2.739
Beta-naphthoflavone	0.032	1.055
Aurone	1.007	1.054
Xanthoangelol	2.781	8.566
Morachalconea	1.285	1.929
Gemichalcone	3.562	9.636
Desmethylxanthohumol	2.169	8.286
Bavachalcone	1.623	6.879

Chlorogenic acid	1.2	1.688
4-Hydroxyderricin	2.123	8.572
Alloimperatorin	1.752	3.047
Xerantholide	1.796	3.221
Xanthogalenol	2.19	8.486
Xanthoangelol E	4.438	8.863
Xanthohumol	3.557	7.575
11-Beta-13-Dihydrolactucopicrin	2.433	7.088
Andrographolide	2.149	6.74
Isoliquiritigenin	0.023	1.109
Butein	0.966	1.16
Nardosinone	2.036	4.713
1,7-Bis-4-hydroxy-3-methoxyphenyl-1,4,6-heptatrien-3-one	0.692	9.571
Sappanone A	0.632	1.44
3'-Geranyl-2',3,4,4'-tetrahydroxychalcone	2.717	9.689
Horminone	1.68	6.787
Kuraridin	1.82	2.916
Magnolioside	1.978	7.792
Calebin A	1.739	2.708
Bergamottin	1.991	6.449
Parthenolide	2.249	4.533
Alantolactone	1.591	2.575
4-Phenylcoumarin	0.02	1.172
XHY-1	2.331	4.256

Table S3 RMSD value of compound docking with Cys151

Compounds	RMSD l.b	RMSD u.b
Gedunin	2.578	4.592
Gemichalcone	4.703	7.297
Caffeic acid phenethyl amide	0.044	1.055
Aurone	1.199	2.065
3'-Geranyl-2',3,4,4'-tetrahydroxychalcone	1.399	2.353
Xanthoangelol	1.348	2.474
Farnesyl Caffeate	5.849	8.658
5'-Prenylxanthohumol	1.438	4.825
Phenylethyl caffeate	0.096	1.058
3-(Phenylmethylene)indolin-2-one	8.841	10.423
4-Phenylcoumarin	1.71	3.186
Licoagrochalcone A	2.451	3.118
Coniferyl ferulate	7.578	9.307
Bavachalcone	1.025	1.698
Auraptene	6.137	8.246
1,7-Bis-4-hydroxy-3-methoxyphenyl-1,4,6-heptatrien-3-one	2.336	5.85
Chlorogenic acid	1.212	1.84
4-Hydroxyderricin	1.876	4.018
Morachalconea	2.832	3.562
Xanthogalenol	5.932	9.315
Desmethylxanthohumol	6.018	9.412
Diosquinone	6.112	7.837
11-beta-13-Dihydrolactucopicrin	1.759	4.126
2',4'-Dihydroxy-6'-methoxy-3',5'-dimethylchalcone	0.258	1.062
Fraxetin	0.836	4.638
Xanthohumol	2.616	3.424
Curcumin	3.625	4.29
Shogaol	2.04	2.47
Xanthoangelol E	2.618	3.459

Sappanone A	8.724	9.661
XHY-1	2.251	6.474
Octylcaffeate	3.361	4.564
Bergamottin	3.47	5.627
isoliquiritigenin	8.864	9.912
butein	9.113	10.153

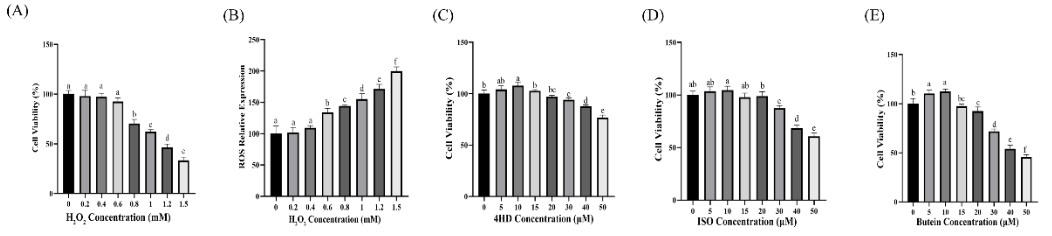

Figure S2. Cell redox levels. (A)-(B) Effect of H_2O_2 on cell viability and ROS level. (C)-(E) Effect of 4HD, ISO and butein on cell viability in HepG2 cells.
